# EFFECTS OF HEARING INTERVENTION WITH HEARING AIDS ON PHYSICAL FUNCTION: FINDINGS FROM THE ACHIEVE STUDY

**DOI:** 10.1093/geroni/igae098.0613

**Published:** 2024-12-31

**Authors:** Wuyang Zhang, Jennifer Deal

**Affiliations:** Johns Hopkins University, Baltimore, Maryland, United States; Johns Hopkins University, Baltimore, Maryland, United States

## Abstract

Hearing loss is an underrecognized risk factor for physical function decline that may be amenable to rehabilitative intervention. Using data from the Aging and Cognitive Health Evaluation in Elders (ACHIEVE) study, a randomized controlled trial of best practices hearing intervention on cognitive decline (Clinicaltrials.gov Identifier: NCT03243422), we investigated the effect of hearing intervention versus health education control on 3-year changes in physical function, a pre-specified outcome for the trial. 977 participants were randomized 1:1. The primary outcomes for this analysis included the Short Physical Performance Battery (SPPB), rescaled according to published guidelines developed in the Health Aging and Body Composition study to address ceiling effects in high-functioning populations, and grip strength. At baseline, the study sample had an average SPPB score of 1.80±0.34, with a mean age of 76.8±4.0 years, 54% female, and 11% self-reported Black race. In linear mixed effects models adjusted for participants’ baseline socio-demographic characteristics, health status, and hearing status (and corresponding time interactions), 3-year changes (in SD units) were did not differ between treatment groups in SPPB scores (β=-0.001; 95%CI: -0.115, 0.113; p-value: 0.98) and grip strength (β=0.016; 95%CI: -0.054, 0.085; p-value: 0.66). In pre-specified sensitivity analysis, the strongest estimated effect of hearing intervention on SPPB performance was among those with worse cognitive test performance (β=0.237; 95%CI: -0.019, 0.493; p-value: 0.07). Given that physical function was not the primary outcome of the ACHIEVE trial, these results should be considered exploratory. Follow-up of ACHIEVE participants is ongoing to determine if intervention effects require longer follow-up time.

